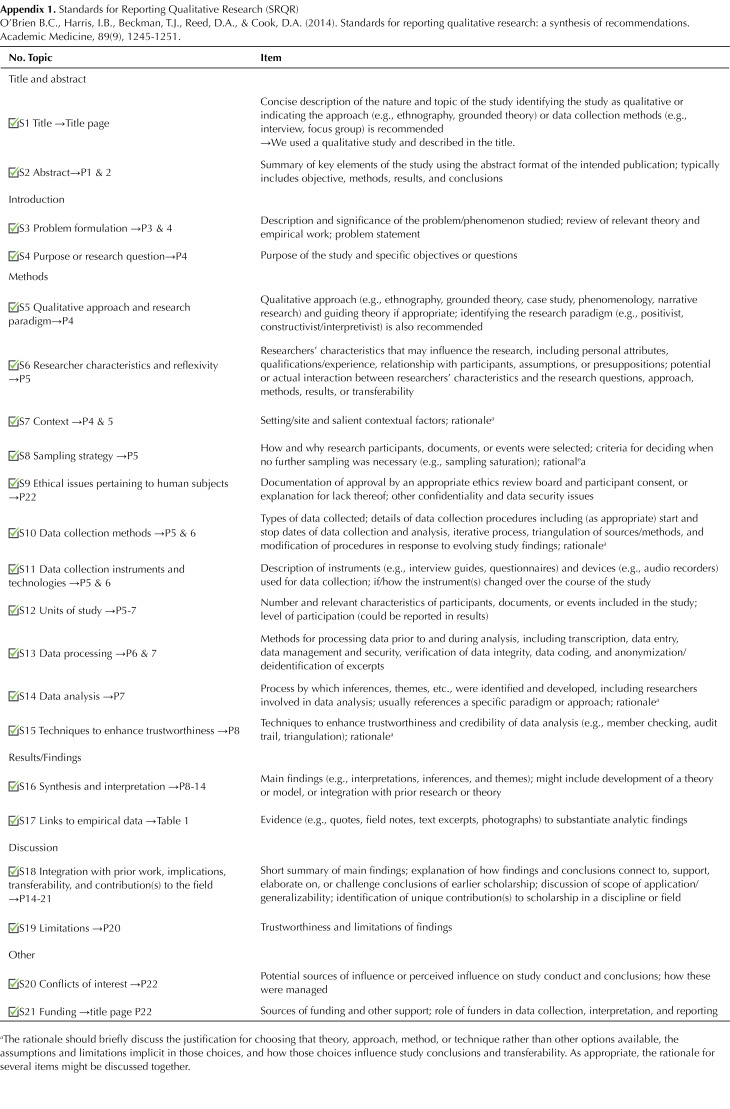# Understanding the process for developing sleep disorders among Japanese workers: a qualitative study

**DOI:** 10.34172/hpp.2021.12

**Published:** 2021-02-07

**Authors:** Ayako Toyoshima, Michiko Moriyama, Hidehisa Yamashita, Md Moshiur Rahman, KATM Ehsanul Huq, Yasmin Jahan, Kana Kazawa

**Affiliations:** Graduate School of Biomedical and Health Sciences, Hiroshima University, Hiroshima, Japan

**Keywords:** Sleep disorders, Workers, Psychosocial factors, Occupational health

## Abstract

**Background:** Sleep disorders have an enormous impact on occupational health and are counterproductive from an economic perspective. However, the processes of causing sleep disorders from psychosocial aspects have not yet been known. The purpose of this study was to describe how sleep disorders develop among workers with respect to different psychosocial conditions.

**Methods:** A conventional qualitative content analysis was conducted with a semi-structured interview among twenty-seven workers (14 males and 13 females) who were diagnosed with sleep disorders or had a self-reported history of sleep difficulties. Study participants were recruited from a specialized clinic and communities using snowball sampling. This paper adhered to the Standards for Reporting Qualitative Research (SRQR) checklist.

**Results:** The results showed that there were four steps involved in the sleep disorders development process. Firstly, participants with sleep disorders developed ‘early warning signs’ with 11 categories of triggers; secondly, ‘aggravating factors’ on top of these early warning signs; thirdly, workers tried to ‘cope with’ their sleep disorders in the ways they thought would be effective. Finally, when coping failed to improve the quality of sleep, it led to the onset of sleep disorders.

**Conclusion:** The development of sleep disorders and triggers of psychosocial factors were revealed. An occupational health nurse can bring these findings in practice for preventing worker’s sleep disorders.

## Introduction

The global prevalence of sleep disorders was recorded from countries ranging from 1.6% to 56.0%.^1^ Sleep disorders have been associated with reduced working performance,^2^ negative physical and mental health outcomes and increased incidence of adverse safety outcomes.^3^ Among the Organization for Economic Co-operation and Development (OECD) countries, economic loss due to sleep disorders is estimated to be between 1.4 to 2.3% of the gross domestic product (GDP).^4^ For these reasons, sleep disorders are considered as an important issue in occupational health.

Several occupational factors are known to interfere with sleep. Studies reported the main factors such as shift work, rest-duration, loneliness, workplace violence, psychosocial stress,^5^ poor discretionary power over their own work hours,^6^ and harassment^7^ were altering the quantity and quality of sleep and the consequences, and suggested health promotion measures.^5-7^

In Japan, particularly long working hours,^8^ high consumption of alcohol, and the shortest sleep time among 10 countries have been reported the increase in higher health risk of workers including sleep disorders.^9^ A cohort study among Japanese employees from various occupations notified that there was an association between workaholism and poor sleep quality in terms of sleep latency and daytime dysfunction.^10^

Even though health professionals in occupational health settings have highlighted the importance of providing information about sleep,^11,12^ specific guidelines on how to prevent sleep disorders among workers are yet to be fully developed. A combination of various practices and habits of sleep hygiene is essential to ensure the quality of a good night’s sleep and full daytime attentiveness.^13^ Workplace-based employees’ health interventions also suggested yoga, physical activities and cognitive-behavioral therapy for insomnia interventions.^14^ However, until now, sleep hygiene is the major intervention at a workplace for the management of sleep disorders.^15^

Moreover, we can easily understand that there are several factors other than occupational environment affecting sleep disorders such as personal issues of family and human relationships. Balancing childbearing, housework, and caregiving along with work are known as stressful situations worldwide.^16^ Japan is ranked as one of the lowest countries in terms of home-task sharing.^17^

Occupational health nursing is responsible for the prevention of diseases related to the work environment and to promote employees’ health.^18^ It also can help employees solve the problems affecting their sleep disorders, when the distinctive physical, psychosocial, and environmental situations are specified. In fact, there has been no qualitative discussion on how sleep disorders develop among workers. If different situations under which workers suffering from sleep disorders were described, it might help to establish strategic ways for preventing these sleep disorders from developing in the occupational health field. The processes of developing sleep disorders from psycho-social aspects have not yet been known. Therefore, it is necessary to figure out those factors and timings of prevention. The purpose of this study was to explore the process of developing the sleep disorders among the Japanese workers and to identify various factors behind these disorders.

## Materials and Methods

### Study design and study setting

Between September and December 2015, we carried out a conventional qualitative content analysis with a semi-structured interview to investigate sleep disorders among the Japanese workers in Hiroshima city, Japan. This study adhered to the Standards for Reporting Qualitative Research (SRQR, Appendix 1).^19^The presentqualitative study was designed to identify the important and appropriate research questions focused on revealing the who (Japanese workers), what (sleep disorders), why, and how they developed sleep disorders, and receiving insights from informants regarding an unclear phenomenon.

### Participants and procedures

Participants were full-and part-time workers, self-employed or employed by private/public organizations, aged ≥20 years. The inclusion criteria for participants were having a diagnosis and receiving treatment for sleep disorders, or a self-reported history of sleep difficulties. Students and the unemployed were excluded from this study.

Participants were recruited from a specialized hospital outpatient clinic and communities. In the hospital, the psychiatrist in charge of sleep disorders introduced the participants to the first author [the principal researcher (PR)] who was a nursing graduate student specialized in occupational health. In the communities, a snowball sampling method was used^20^ in which the PR found two informants who met the inclusion criteria and asked them to introduce other workers through their networks. The PR conducted individual face-to-face in-depth and semi-structured interviews lasting approximately 60 minutes, and these were conducted either once or twice when further information was needed. To maintain privacy, the interviews were conducted in a clinic or in an independent PR’s office. Researchers stopped recruiting participants when all the different patterns of sleep disorders among patients [reference related to the international classification of sleep disorders-3 (ICSD-3)] and all causes of their sleep difficulties were saturated.

A total of 27 participants (14 males and 13 females; age between 20 to 60 years) were recruited from the hospital and the communities. The pattern of sleep disorders among the participants was explained in Table 1.

### Data collection tool

Participants were asked to reflect on the experience of their sleep disorders. They were asked to answer the following questions: (1) How did you perceive your physical and mental health when you began to have a trouble in sleeping? (2) How was your social situation at that time? (3) Did you have any subjective symptoms? If your answer is ‘yes’, what were the symptoms and what were they like? (4) Did your sleeping difficulties affect your business? If your answer is ‘yes’, how did it affect your business? If your answer is ‘no’, please tell us how you were able to manage your business without being affected by your sleep disorders. (5) Why and how did you cope with sleeping difficulties?

Participants’ characteristics such as gender, age, occupation, past medical history, and current medical condition were also collected. A digital voice recorder was used to record the interviews after obtaining consent from the participants.

### Data analysis 

The principal researcher performed a conventional content analysis for this study. (1) The contents of the interviews were documented and used for analysis. The analysis findings were extracted, and categorized responses related to the sleep disorders development process, (2) the reasons for developing or not developing a coping behavior, and (3) assessed the impact of sleep disorders had on work.

We used ICSD-3^21^ to categorize major sleep disorders such as insomnia, sleep-related breathing disorders, central disorders of hypersomnolence, circadian rhythm sleep-wake disorders, parasomnias, sleep-related movement disorders, and other sleep disorders. When the participants in the community had no diagnosis by a specialist, on the basis of the Algorithm for the Evaluation of Chronic Insomnia,^22^ we evaluated the conditions of the patients’ sleep disorders. We asked the participants about their sleep-wake timing patterns, snoring, and restless leg symptoms, etc., and categorized these patterns for the purpose of our study.

### Ensuring trustworthiness and credibility 

All the data were carefully analyzed on the meaning of the contents, were coded, and categorized by researchers who specialize in occupational health, chronic care and qualitative analysis. In addition, collaborators who were psychiatric specialists and sleep experts at the hospital confirmed the validity of the analysis contents. Data were also checked by the research participants, and they were requested to confirm the consistency and credibility of the contents.

## Results

### Process of sleep disorders development 

**Figure 1:**
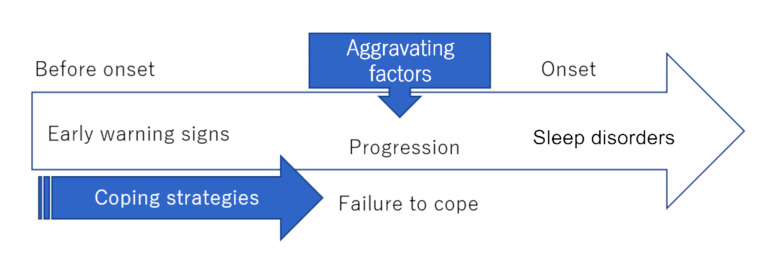
Process of developing sleep disorders

Participants were requested to state what they thought triggered the development of their sleep disorders and what they did to overcome it. Four steps were generated for developing sleep disorders. First, they developed ‘early warning signs’ before starting to experience sleeping difficulties. Secondly, ‘aggravating factors’ were added to these ‘early warning signs’. Thirdly, workers tried to ‘cope with’ sleeping difficulties. Lastly, when the coping strategy failed to resolve their sleep difficulties, this led to the onset (‘impact on work’) of their sleep disorders. However, they did not develop a sleep disorder when the coping strategy was effective because it made them sleep well (Figure 1).

*Early warning signs*

The main category (phase) of ‘early warning signs’ was generated and consisted of 11 categories and 27 subcategories (Table 2).

Early warning signs, which were triggering factors, consisted of physical, psychosocial, and environmental, behavioral, and combined factors along with holding excessive beliefs about sleep that the participants were experiencing or had developed before they began experiencing sleep deterioration or deprivation.

The categories include: (1) ‘Insufficient sleeping hours due to work environment’ and (2) ‘Lack of support resources’. Physical and environmental factors symbolize work culture in Japan characterized by long-commuting distance, long working hours, and the custom of drinking alcohol after work, leading to a lack of sleep. A participant mentioned, *“Every day, I work until 1am or 2am. If I can go back on the last train, I am lucky. It is the same for everybody.” Another person explained, “On weekends, I have to attend my children’s events and housework that cannot be done on weekdays. My husband just orders me. My parents’ home is far, I cannot ask for help.”*Lack of support was often related to a lack of workload management. At home, gender beliefs regarding housework, and long working hours at the company and side jobs, due to economic conditions had an influence on this. (3) ‘Jet lag and social jet lag’ is physically produced circadian rhythm imbalance by workers’ frequent overseas business trips, shiftwork, and wide differences between workdays and off days sleeping time.

(4) ‘Disease symptoms’ such as uncontrolled itchiness and (5) ‘Pregnancy/Breast feeding’ as a physical factor that deprived them of a good night’s sleep. (6) ‘Caregiving’ and (7) ‘Ill family members’ were physical and psychological factors that also disturbed sleep. A participant stated, *“My father-in-law hurt me by bad words. I struggle, should I take care of him? It sticks my heart and cannot sleep.”* Another participant spoke, *“After my father’s health got worse, I was worried whether he was alive at night, so I woke up and went to check on him many times. It lasted for months.”* Caregiving at home generates a physical burden and can cause conflict with the carer. Negative emotions such as anger and worry often disturb their sleep over a long period of time. (8) ‘Family conflict’ and (9) ‘Personal conflict’ were psychological factors displaying negative feelings towards family member(s) and/or his/herself and were difficult to control. A participant stated, *“I was always told to take over the family business, but I did not have the qualification. I always suffered from an inferiority complex.”* Another participant mentioned, *“My child came back to my house with her children. Younger ones have their own time. I got to sleep late at night too, but I have to wake up first and wake everyone up.”*We observed the feeling of inferiority complex among family member(s), worries concerning unfinished business, an uncontrollable time schedule for family members and feeling of anger. The presence of long-term personal conflict also deprived them of sleep.

Stereotypes regarding sleeping time and false knowledge of drowsiness were categorized as (10) ‘Misbeliefs about sleep’. Some participants obsessively attached high importance to their bedtime routine, i.e.* “I am a person who cannot sleep before 12 midnight.” “I need 7 hours sleep, but when I couldn’t then I really get upset.”* As a behavioral factor, (11) ‘Habits of behavior’ were extracted. This includes thinking about or checking the Internet in bed, drinking alcohol before bed, sleeping during the daytime, and so forth.

Nobody stated about their physical environment such as noise, lightning, temperature, bed or mattress, which was related to the exacerbation.

*Aggravating factors*

An aggravating factor is another main category generated from the interviews. As early warning signs were not resolved and persisted, several factors have been attributed to as factors aggravating sleep conditions. From 19 subcategories, 8 categories were generated. They included (1) ‘Accumulation of fatigue’ in which people continued to sleep for short hours, a hyperactive condition continued, and some reported a drop in their physical strength. (2) ‘Feeling of urgency’ comes from continuous intensive work and upset them. (3) ‘Crisis of social survival’ is when they felt fear of losing their position and caught up in feelings of defeat amid the competitive environment at work. (4) ‘Life events and loss’ included loss of family members, someone close to the participant, familiar workplace, work position, and meaning of work. (5) ‘Changes in work’ refers to changes in working shifts and being unable to adjust to new tasks. (4) or (5) could be a trigger itself, but in this intervention these factors appeared after the warning signs. (6) ‘Guilt for absence’ refers to increased feeling of guilt because they needed to be absent due to their physical condition. (7) ‘Aggravation of physical condition’ is an aggravation of tachycardia; these physical conditions made their concerns worse. (8) ‘Economical anxiety’ results from a combination of age, single status (being unmarried), health conditions, and an obsessive for survival, all escalating their anxiety.

The balance between physical ability and amount of work became worse because of a decline in physical ability and insufficient sleeping time to recover from fatigue. The participants felt pressured in the workplace and at home from deadlines or when responding to emergencies. These feelings aggravated their sleeping difficulties.

They became worried that they would lose their job, and they had an inferiority complex with colleagues or worried that they might lose their role at the workplace because of their low performance evaluation. This leads to insomnia as they have difficulty adapting to the changes in new context or different working hours. In particular, bereavement and change of workplace make them more vulnerable and cause grief as they feel loneliness and separated from their family.

*Coping strategies*

The third main category generated from the interviews is ‘coping strategies’. It consisted of 2 categories i.e. (1) ‘getting support for solving their problems’, which includes support from private such as family and friends, and official services such as harassment consultation, nursing services, and medical support. (2) ‘Personal efforts for self-care’, which includes taking sleep aid supplement, relaxation, maintaining sleep hygiene behavior, and lifestyle changes. Some participants did not ask for help because of reluctance to visit a psychiatry clinic, taking medication, fear of retaliation after appealing to public services, and obsessive thoughts about mother/caregiver and being unable to request for aid.

*Impact on work*

The fourth main category generated from the interviews is ‘impact on work’ as a result of all processes. The consequences of sleep disorders on work were categorized into two; one was the ‘negative impact on work’, and the other one was the ‘able to do routine work’ with no impact on work. In the negative impact, participants reported that insomnia symptoms such as declining concentration or strong drowsiness had a negative impacted on their business. On the other hand, some could do the routine work despite having insomnia symptoms. They felt they could do their job without making mistakes or causing troubles when they did the routine work.

*Sleep disorders other than insomnia and circadian rhythm disorders*

In this study, sleep apnea, central hypersomnia, parasomnia, and sleep-related movement disorder were found the different processes from insomnia and circadian rhythm disorders. We did not observe any early warning signs or aggravating factors. Awareness of mental and physical disorders due to drowsiness during the daytime was recognized but because of minimum knowledge about these sleep disorders, nobody perceived any threat or necessity to seek treatment. Therefore, the factors that triggered them to take action were: when their family brought these factors under their notice, or at their workplace, they were recommended to go for a medical examination, and found out, when they were exhibiting physical symptoms or diseases such as obesity, diabetes, and depression. They responded that they did not know whom they should consult, did not feel the necessity to undergo examination or treatment, interruption of medical treatment, and they could not lose weight in case of obesity.

## Discussion

This study aimed to find out the process of developing sleep disorders and identified underlying factors and its impact on work. We believe a holistic approach is necessary with respect to occupational nursing perspective, paying attention to not merely on working environment and sleep hygiene, but also, emphasis on psychosocial factors to intervene. Nursing is a medical profession, and it is important to strengthen the services and inter-professional co-operation in healthcare. This study revealed that Japanese cultural practices of long-working hours, drinking customs after the work, and burden of housework reduce the total sleep duration which influenced to initiate the early warning signs of sleep disorders. Sleep patterns differ among countries influenced by sociodemographic and cultural status.^23^ In our study, we found that sleep disorders were related to physical, psychosocial, environmental, behavioral, and combined factors. Similar findings were observed that sleep disorders increased with medical and psychological comorbidities.^24^ Another study documented that sleep disturbance has a significant association with physical, psychosocial, and environmental factors.^25^ This study also described the types of features as participants’ personal, family and working environment can influence to develop sleep disorders.

Sleep has a vital role on various body systems including brain functions. Our results indicated that occupational health services, like sleep hygiene education or personal counseling might be effective for improving the quality of workers’ sleep when they displayed some early warning signs and aggravating factors. Many occupational factors may interfere with sleep which may cause significantly short and long-term effects on health and safety.^26^ In Japan, occupational factors such as higher work demands, longer working hours, shift work, etc. are individually associated with sleep disturbance.^27^ Similarly, some other Asian countries such as South Korea documented that violence, discrimination, work-life imbalance, job dissatisfaction, high work demands and intensity, and job insecurity are the common responsible factors for sleep disturbance.^28^ The current study identified some responsible factors on how workers develop sleep disorders like long working hours, caregiving practice, housework, nurturing baby, jet lag, social jet lag, some disease conditions (i.e. itching, pain), family or personal conflict and behavioral habits. Several studies found that jet travel and night work resulted in a huge change at the time for sleep and wake, a large phase shift, producing circadian misalignment between sleep, work, meals and the internal circadian rhythms.^29,30^ The high job demands and overtime are important factors for long working hours that have been associated with sleep disturbances.^27^ However, long working hours and sleep complaints might be partially accounted for behavioral correlates like a higher level of Internet addiction, alcohol consumption and activities tended to have poorer sleep quality.^31^ The negative psychological and physiological impacts of inadequate sleep also have been well-documented, including emotional incidents,^32^ and increased risks of participating in hazardous behaviors (e.g., tobacco and/or alcohol consumption, driving while under the influence of insufficient sleep).^33^ Based on the aforementioned discussion, studies recommended developing a policy for scheduled naps and providing a quiet room for rest breaks and naps can be a countermeasure to reduce fatigue, which can provide benefits to the workers.^34^

In this study, we described categories of early warning signs and aggravating factors and how workers develop sleep disorders. Some aggravating factors such as accumulation of fatigue, urgency, and feelings of guilt for being absent from work due to chronic diseases were related to work-life balance. The most common aggravating factor was fatigue for workers, especially those who were doing a shifting duty. Shifting duties of the workers reduced the quality and quantity of sleep that negatively interrupted their quality of life and health including social activities.^35^ This study indicates psychological stress due to caregiving (taking care of ill family members) and housework without surrounding supports can also cause fatigue. There are two key contributors to develop fatigue, insufficient sleep and disruptions in the normal sleep cycle cause to circadian misalignment. Tanaka et al. reported that alterations in the biological rhythm increased circadian biological dysfunctions, such as energy metabolism, autonomic activity, endocrine, and neurocognitive dysfunctions.^36^ It is well documented that chronic illness such as cardiovascular disease, diabetes, rheumatoid diseases, and respiratory disorders are associated with reduced health-related work performance and poor quality of working life after the disease onset.^37,38^ Similarly, lack of quality sleep can negatively impact work performance, attention to tasks, and decision-making factors that may increase workload and work-related stress.^39^ In addition, discrimination and prejudice at the workplace which have been associated with emotional stress disproportionally affect workers with chronic diseases.^40^ These results suggest the need to promote proper working hours and the need for workers to have time to refresh. These findings also demonstrate the importance of a good balance between the treatment of chronic diseases and working. From the above discussion, it appears that individuals would be benefited from those aggravating factors by a multi-disciplinary approach that helping the integration process that encompasses the physical, emotional, social, vocational, and existential work of chronic illness adjustment. Moreover, rehabilitative programs, health promotion programs and more involvement from health professionals, particularly nurses, have been advocated as potentially important services for adults with chronic illness to learn to optimize life.^41^ Based on the above results, addressing the causes of early warning signs and aggravating factors, it may become possible to construct a program to prevent the onset of sleep disorders for employees who suffer in the occupational health field. In occupational health settings, workers with sleep-related support do not always require diagnosis at a medical institution. It is necessary that a worker who has difficulty in sleeping, regardless of the presence or absence of a sleep disorder-related diagnosis, should be provided with occupational support in order to prevent developing sleep disorders.

Furthermore, we explained why participants were unsuccessful with coping strategies before developing sleep disorders. Some participants did their best given their own situation and tried to improve their lives such as by adapting to a new job or family environment. It confirmed that non-pharmacologic management such as sleep hygiene education, counseling, and introducing social resources in occupational health would be helpful as an effective coping strategy against sleep disorders. The highly stressed workers may be more prone to sleep disturbance, nightmares, daytime malfunction, and lack of rest due to sleep deprivation. In this regard, appropriate stress coping strategies can improve sleep disorders even in the most highly stressed individuals.^42^ Effective coping styles that involve actively engaging problems, positively interpreting situations, and using humor lead to faster resolution of difficulties and help maintaining the psychological health of an individual during times of stress, thereby allowing a greater sense of safety and security. Coping could be either problem-focused (e.g., by employing problem solving and time management strategies) or emotion-focused (e.g., through mindfulness, relaxation, and obtaining emotional support from colleagues or friends).^43^ So, the appropriate strategy should be implemented to reduce work stress and sleeping disorders from four main coping strategies which have been discussed elsewhere.^44^

Our findings revealed that workers with sleep disorders are likely to have a negative impact of low performance at work or be absent from work. This is in line with a previous study corroborating that sleep disorders are costly health problems and resulting in the high estimated costs to society of leaving the most prevalent sleep disorders untreated that would be incurred by providing effective management.^45^

The study has several limitations. The participants were recruited through a snowball sampling method in the community, so selection bias is a concern. The study participants were also selected from a single city in Japan which can restrict the generalizability of the study. During the data collection, some participants were asked to tell the previous history related to their sleep disorders, so recall bias is another concern.

### Implications for occupational health nursing practice

In Japan, there is a major change in the labor force due to increasing aged population. As a consequence, Japanese occupational health nurses adopted dramatically in recent years to handle new and changing risks among workers.^46^ Recently, companies adopted a good work-life environment such as short working hours for childbearing women and stop caregiving for all workers. However, still there are many issues to pay attention as an occupational health nurse.

The findings of this study can be used for a program to manage sleeping disorder for workers. We have developed an educational program and booklet and applied it to company workers, in which we listed all factors generated from this study and added assessment tools and instructions on how to deal with those factors specifically. We also described the process of sleep disorders development. In the booklet, we brought attention to the early warning signs before the onset. As an occupational health nurse, not only providing sleep hygiene information, we can pay more attention to these individuals and environmental factors and can facilitate in case management and collaborate with companies to deal with the work conditions. The educational program and materials we developed based on this study could be widely implemented nationally and internationally for further evaluation by the occupational health nurses.

## Conclusion

Among the study participants, nobody had the knowledge about the category of sleep disorders. There are a certain number of participants who had no understanding of their sleep difficulties such as SAS and other sleep disorders. They could not recognize, did not ask for supports, did not follow coping strategies, and tried to deal with disorders by themselves. Therefore, routine screening system to evaluate workers’ sleep condition by using standard tools/questionnaires, counseling and treatment are essential. It is also important to know the types of sleep disorders which allow accurate diagnosis, improved communication with a sleep specialist, and standardization of management plan to improve the quality of life. This study suggests a holistic approach involving both workers and occupational health nurses for the prevention and control of sleep disorders.

## Acknowledgements

The authors would like to thank all participants who shared their experiences and perceptions in this study.

## Funding

This study is carried out with funding of the Grants-in-Aid for Scientific Research Program (KAKENHI), Japan (No. 15H05078).

## Competing interests

The author(s) declared no potential conflicts of interest with respect to the research, authorship, and/or publication of this article.

## Ethical approval

The study protocol and procedures were approved by the ethics committee of Hiroshima University, Japan (E-80). Written informed consent was obtained individually from all the participants.

## Authors’ contributions

AT and MM were responsible for the conception and design of the study. AT involved in data collection. AT, MM, HY, and KK worked on data analysis and interpretation. AT, EH and YJ drafted the original manuscript. MM, HY, MMR and KK critically revised and finalized the manuscript. All authors read and approved the final manuscript.

**Table 1:** Characteristics of participants

**Age category**	**Gender**	**Occupation/Job type**	**Types of sleep disorders**
40's	Male	Office work	Insomnia
50's	Male	Office work	Insomnia
40's	Male	Factory work	Insomnia
60's	Female	Nurse (no shift work)	Insomnia
60's	Female	Elementary school teacher	Insomnia
40's	Male	Agriculture	Insomnia
60's	Female	Delivery work	Central hypersomnia
40's	Male	Office work	Insomnia
60's	Male	Office work	Parasomnias
40's	Male	Office work	Insomnia
50's	Male	Technologist	Insomnia, sleep apnea syndrome
30's	Female	Office work	Central hypersomnia
30's	Female	Nurse (no shift work)	Insomnia
50's	Female	University faculty	Insomnia
30's	Female	Nurse (no shift work)	Insomnia, sleep related movement disorders
60's	Male	Office work	Insomnia
30's	Female	Nurse (no shift work)	Insomnia, circadian rhythm disorder
40's	Male	Office work	Insomnia
40's	Male	Manager	Insomnia
40's	Female	Nurse (no shift work)	Insomnia, sleep apnea syndrome
40's	Female	Professional	Insomnia
30's	Female	Nurse (shift work)	Insomnia
50's	Male	Technician	Insomnia
20's	Female	Nurse (childcare leave)	Insomnia
60's	Female	High school teacher	Insomnia
60's	Male	Office work	Sleep apnea syndrome
50's	Male	Medical technician (with night shift)	Sleep apnea syndrome, sleep related movement disorders

**Table 2:** Onset of early warning signs for sleep disorders

**Category**	** Subcategory**	**Code**
Insufficient sleeping hours due to work environment	Workplace habits of working until midnight	Long hours labor
		Working environment to work until midnight
		Unable to finish work duty within working hours
	Long commuting hours	Commuting time reduces sleeping time
	Workplace with habit of drinking and eating out after work	Drinking and eating out after work leads to going home later
		Workplace drinking party customs
Lack of support resources	Poor surrounding support resources	No one to help with the job and consult sleep difficulties in the workplace
		No one to help with housework and childcare
Jet lag and social jet lag	Overseas business trip	Using sleeping pills to adjust to the time difference when traveling abroad
	Shift work or time difference work	Night work
		Do not know how to adjust one’s biorhythms during night shift
	Inconsistent waking time because the schedule depends on the workload
	Sleeping on weekends	Weekends used to catch up on sleep missed on weekdays
Delayed sleep-phase due to sleeping in	
Disease symptoms	Uncontrolled symptoms	Uncontrollable symptoms such as itchiness by atopic dermatitis, pain, cough due to asthma
Pregnancy/Breast feeding	Breast feeding	Children crying throughout the night
		Breast feeding at night
	Pregnancy	Morning sickness
Caregiving	Physical and Mental fatigue	Worries about family members with dementia wandering around and often need to go and find her/him
		Frequent trips to the toilet during the night
	Conflict with a carer	A carer throws/shouts unpleasant words. Cannot allow it.
	Beliefs about use of social resources	Taking care of family member is family’s task. Cannot ask for help. Use of social resources is not accepted.
Ill Family members	Prolonged anxiety and nervousness (tension) for anticipating changes in family’s situation	Worried about whether the family is alive
		Nervousness due to unstable family condition (take care of the family at bedside, and so nervous)
	Anticipatory grief	Anxiety about living after family dies
Families conflict	Anger, inferiority complex for families	Inferiority complex that a family business cannot be inherited
		Loneliness in the home, suppression of anger
	The pace of my life is disturbed by adjusting time to fit in with the lives of others	Always adjusted my time to family time
Personal conflict	Keeping complaints inside/unable to solve problems directly	Too much stress at work/human relationships, but cannot tell anybody, cannot ask for help/ cannot resolve the problem
	Unfinished business	Unfinished developmental tasks Unmarried / pregnancy age limit
	Bad handling of emotions	Cannot handle emotions well. Anger and complaints inside.
Misbelief about sleep	Excessive commitment to sleeping time	Commitment to bedtime routine
		Commitment to total sleeping hours
	Obsession/misunderstanding about their sleep pattern	Cannot sleep well since a child. I am a bad sleeper. It takes a long time for me to fall asleep.
	Wrong knowledge about sleep	Once missed the chance to fall asleep, cannot sleep anymore.
Habits of behavior	Thinking in bed	Thinking deeply in bed
		Reflecting on the day in bed and arranging tomorrow’s business. Also, deep regrets about the day and cannot sleep.
	Coping behavior to relief stress	Internet, smart phone in bed
		Drinking alcohol before bedtime
		Eating late at night and before bedtime
	Exposure to information until just before bedtime	Checking the latest information on the bed
	Difference of sleep pressure peak and living conditions	Allowing them to sleep when they most want to sleep (Sleep in daytime/ right after coming home/ after dinner, so cannot sleep at night).

**Appendix 1:**